# Dark Blood Imaging of the Heart using Dual-Inversion, Balanced Steady-State Free-Precession

**DOI:** 10.1186/1532-429X-18-S1-P306

**Published:** 2016-01-27

**Authors:** Marcos P Ferreira Botelho, Ioannis Koktzoglou, Wei Li, Robert R Edelman

**Affiliations:** 1 Radiology, Northwestern University, Chicago, IL USA; 2 Radiology, NorthShore University HealthSystem, Evanston, IL USA

## Background

Dark blood images of the heart are typically acquired using an ECG-gated, single-slice, dual-inversion turbo spin-echo (TSE) pulse sequence. We hypothesized that a dual-inversion balanced steady-state free-precession (DB-bSSFP) pulse sequence might provide more efficient multi-slice coverage with less sensitivity to cardiac motion.

## Methods

The study was approved by the institutional review board and written, informed consent was obtained. Six healthy subjects were imaged at 1.5 Tesla and 3.0 Tesla (MAGNETOM Avanto and Verio systems; Siemens, Erlangen, Germany). The DB-bSSFP sequence used a two-shot bSSFP readout with duration of ~150 msec. The sequence was triggered to every second R-wave and a chemical shift-selective RF pulse was applied for fat suppression. Both radial and Cartesian trajectories were evaluated. Comparison was made to a standard dual-inversion turbo spin-echo pulse sequence that acquired nine echoes per readout. Image quality was evaluated by a single observer using a four-point scale (1: non-diagnostic, 2: fair, 3: good, 4: excellent) for the LV free wall, septum, RV free wall and RV trabeculae.

## Results

At least four slices could be acquired per breath-hold using the DB-bSSFP technique, compared with only a single slice for DB-TSE. Mean myocardium-to-blood pool contrast-to-noise ratios at 1.5T [3.0T] were: radial DB-bSSFP, 18.2 [22.1]; Cartesian DB-bSSFP, 17.6 [26.3] (p < 0.01); DB-TSE, 14.6 [23.2] (p < 0.05). Over both magnetic field strengths, mean image quality scores for radial DB-bSSFP/Cartesian DB-bSSFP/DB-TSE were 3.8/3.8/3.2 for the LV free wall (p < 0.05), 4.0/3.8/3.5 for the septum (p < 0.05), 3.8/3.8/3.1 for the RV free wall (p < 0.01), and 3.8/3.8/2.9 for the RV trabeculae (p < 0.01). DB-bSSFP showed the least blurring from cardiac motion, particularly noticeable in the LV free wall and septum. Excellent depiction of the thin RV free wall was obtained in all subjects with DB-bSSFP. Radial DB-bSSFP permitted the use of small fields of view without wrap artifact, unlike Cartesian DB-bSSP or DB-TSE.

## Conclusions

DB-bSSFP appears to be a promising, more efficient alternative to DB-TSE for morphologic imaging of the heart.

**Figure 1:**
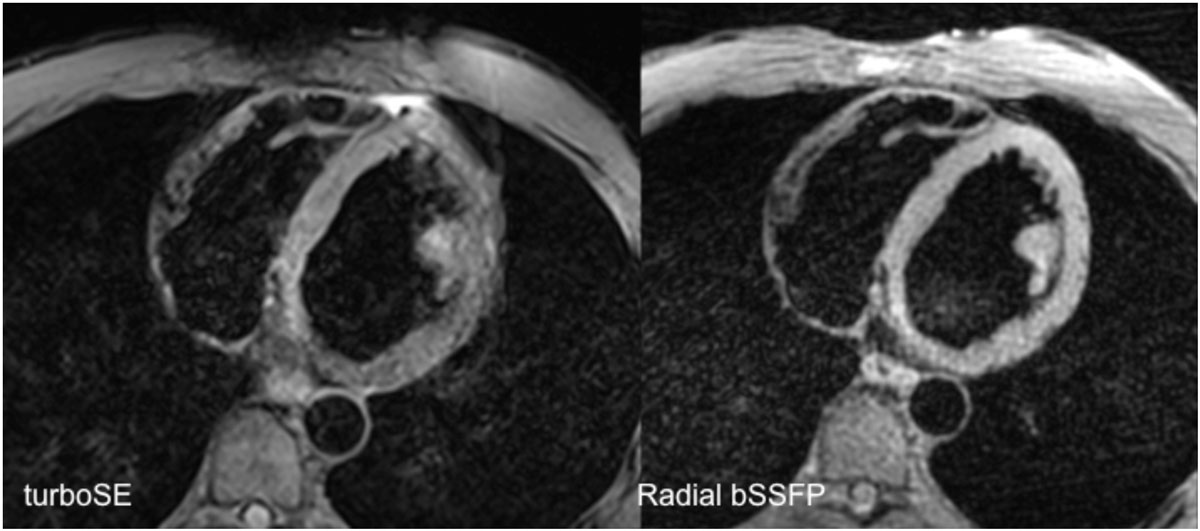
**Dark blood turbo spin-echo (left) shows blurring from motion artifact in the free wall of the left ventricle, not present with dark blood radial bSSFP (right)**.

